# Distal osteotomy of the first metatarsal bone for the correction of hallux valgus: comparison of the sagittal stability of two percutaneous techniques—a cadaveric study

**DOI:** 10.1186/s13018-023-03702-y

**Published:** 2023-03-18

**Authors:** Ester Navarro-Cano, Kerbi Alejandro Guevara-Noriega, Anna Carrera, R. Shane Tubbs, Maria Angeles Sanjuan-Castillo, Joe Iwanaga, Sara Vizcaya, Francisco Reina

**Affiliations:** 1 Orthopedic Surgery Department, Sant Celoni Hospital, Sant Celoni, Spain; 2 Vascular Surgery Department, Hospital Universitari Parc Taulí, Sabadell, Spain; 3 Medical Sciences Department, Clinical Anatomy, Embryology and Neurosciences Research Group (NEOMA), Faculty of Medicine, University of Girona, 77 Emili Grahit St., 17003 Girona, Spain; 4 Department of Neurosurgery, Tulane Center for Clinical Neurosciences, Tulane University School of Medicine, New Orleans, LA USA; 5 Department of Neurology, Tulane Center for Clinical Neurosciences, Tulane University School of Medicine, New Orleans, LA USA; 6 Department of Structural and Cellular Biology, Tulane University School of Medicine, New Orleans, LA USA; 7 Department of Oral and Maxillofacial Anatomy, Graduate School of Medical and Dental Sciences, Tokyo Medical and Dental University, Tokyo, Japan; 8 Department of Neurosurgery and Ochsner Neuroscience Institute, Ochsner Health System, New Orleans, LA USA; 9 Department of Anatomical Sciences, St. George’s University, True Blue, Grenada; 10 Orthopedic Surgery Department, La Linea de la Concepcion Hospital, Cadiz, Spain; 11 Radiology Department, Sant Celoni Hospital, Sant Celoni, Spain

**Keywords:** Foot, Hallux valgus, Minimally invasive, Percutaneous surgery

## Abstract

**Background:**

Distal first metatarsal osteotomy is used to correct mild or moderate hallux valgus (HV). We designed a cadaveric study to compare the resistance to axial load between two percutaneous distal first metatarsal osteotomies: Bösch osteotomy and percutaneous chevron. The first aim of this study was to develop a systematic technique for measuring the sagittal displacement on lateral foot X-rays. Our second objective was to measure the resistance to axial load for both of these osteotomies.

**Methods:**

Ten pairs of freshly frozen cadaveric feet were randomly assigned to one of the two techniques investigated. Pre- and post-operative lateral X-rays were obtained. After surgery, the feet were placed under progressive axial loads up to 60 kg. Metaphyseo-diaphyseal angle (MDA) and the distance between bone fragments were measured, and the differences between the two techniques were statistically assessed.

**Results:**

The MDA decreased in both surgical techniques. The mean plantar tilt was −6.90 degrees (SD = 10.251) for chevron osteotomy and −5.34 degrees (SD = 16.621) for Bösch osteotomy. There was no significant difference between the techniques (*p* = 0.41).

Regarding the distance between the bone fragments, the Bösch osteotomy produced more plantar displacement than the chevron osteotomy, which was statistically significant for the 10 and 20 kg loads (*p* = 0.031 and 0.04, respectively). At loads ≥ 30 kg, the bone fragment distance did not differ significantly between the techniques (*p* = 0.114).

**Conclusions:**

Although the chevron technique confers higher stability regarding fragment displacement during axial loading, both techniques increase the plantar angulation of the metatarsal head.

**Level of evidence:**

Cadaveric study. Level V.

## Background

Percutaneous forefoot surgery to correct hallux valgus (HV) is reported to have advantages over open surgery, such as fewer cutaneous/infectious complications, shorter procedural time, and quicker post-operative recovery [1–3].

Distal first metatarsal osteotomies (DFMO) are normally indicated for treating moderate hallux valgus, with intermetatarsal angle between 12 and 20 degrees [4]. Numerous percutaneous DFMO have been described using different shapes of osteotomy and with or without osteosynthesis [4–6]. Although most authors performing percutaneous DFMO prefer to combine the osteotomy with a cannulated screw [7], some techniques have been described using minimal fixation (Kirschner wire) or no fixation whatsoever [3, 6, 8]. There have been few studies comparing DFMO techniques, as most publications report the results of one specific operative technique [2, 4, 11, 12]. The few comparative studies, published to date, usually compare an open procedure with a percutaneous one [9, 10].

On the other hand, most studies focus only on the anteroposterior (AP) X-ray view; stability in the sagittal plane is seldomly included. This could be due to the difficulty in taking any measurement on the lateral X-ray, as bony profiles might be hard to distinguish. However, as an alteration on the sagittal position of bony fragments can potentially affect the load distribution of the forefoot, it would be desirable to assess sagittal displacement. Recently, weight-bearing computer tomography has facilitated the assessment of the sagittal plane, but many professionals do not have it readily available yet and still rely on plain weight-bearing X-rays.

In the present study, set in an anatomy laboratory, we compared two common percutaneous distal first metatarsal osteotomies. The first is a technique described by Bösch in 1990 [11]. It consists of a linear osteotomy at the metatarsal neck level using a Kirschner wire as a lever to lateralize the metatarsal head. The K-wire does not traverse the metatarsal head, but goes through the capsule of the metatarsophalangeal joint. The second procedure was a percutaneous chevron osteotomy, a V-shaped osteotomy at the metatarsal neck level without hardware stabilization. For both techniques, the authors allowed immediate ambulation using post-operative shoes that transfer weight-bearing to the hindfoot [3]. Even with this shoe, the axial forces acting on the forefoot during full weight-bearing ambulation are still remarkable [13] and could potentially affect non-fixated osteotomies.

The first objective of our study was to develop a systematic and reproducible way of measuring displacement on a lateral foot X-ray. The second objective was to assess the behavior of these two surgical techniques under a controlled axial load.

## Methods

### Study design and specimens

Two percutaneous non-fixated DFMO were compared: percutaneous chevron osteotomy and Bösch osteotomy.

Ten pairs of freshly frozen cadaveric adult feet were obtained from a body donation program of the Medical School of the University of Girona following the legal procedures and ethical framework governing body donation in our country. The authors state that every effort was made to follow all local and international ethical guidelines and laws that pertain to the use of human cadaveric donors in anatomical research [14]. Each specimen consisted of a whole foot and the distal third of the tibia and fibula. Specimens with scars or histories of trauma or surgery were excluded. The age at death of the specimens ranged from 62 to 84 years. Before starting the procedures, the feet were thawed at room temperature.

### Surgical technique

The two surgical techniques were distributed randomly, so that a different technique was applied to each foot in a pair. All surgical procedures were performed under fluoroscopic control with the assistance of a C-arm (OEC Brivo, General Electrics, Boston, Massachusetts, USA).

#### Chevron osteotomy

**Fig. 1:**
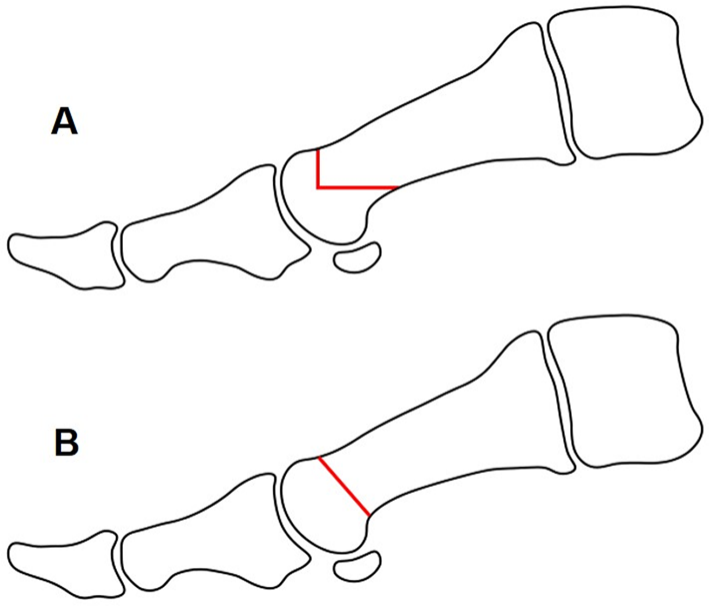
Sagittal view of the osteotomies. **A** Chevron osteotomy: an initial vertical cut is made from the dorsal aspect of the neck of the first metatarsal bone to the middle of the bone in the sagittal plane. The direction of the osteotomy then changes, heading proximally in an almost horizontal direction. **B** Bösch osteotomy: a diagonal linear osteotomy is performed from the dorsal aspect of the neck of the first metatarsal, moving plantarly and proximally

The chevron osteotomy (Fig. 1A) was created using a 2 mm diameter and 15 mm length Shannon burr. It was placed on the dorsal part of the metatarsal at the level of the neck of the bone (junction between the distal metaphysis and diaphysis of the first metatarsal) and a vertical osteotomy was started, moving toward the plantar aspect of the metatarsal. Once the dorsal half of the bone was cut, the direction of the osteotomy changed, heading proximally in an almost horizontal manner. A V-shaped osteotomy was thereby achieved with an angulation of approximately 90 degrees.

#### Bösch osteotomy

**Fig. 2:**
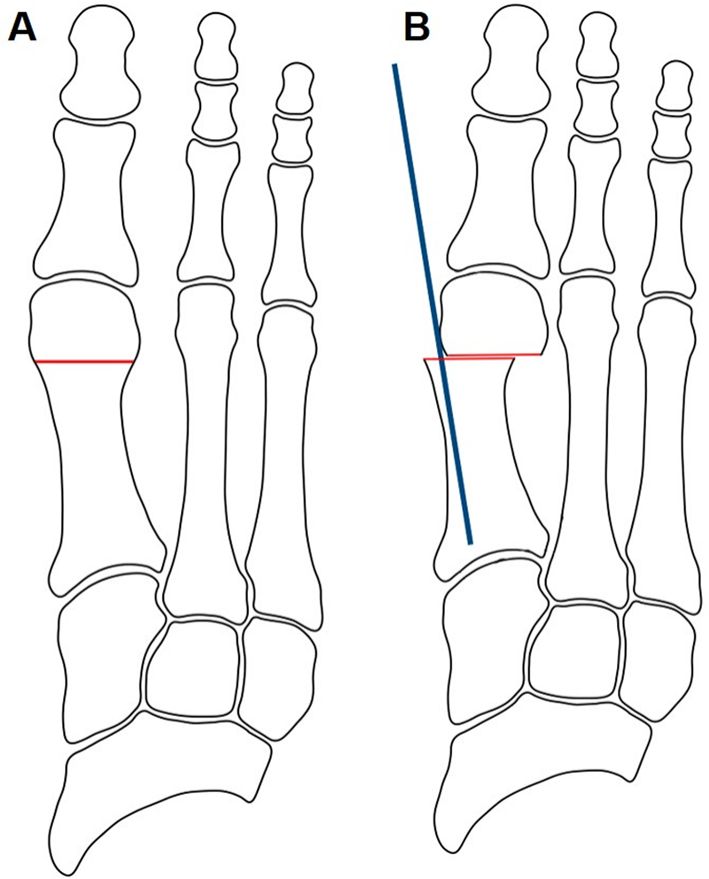
Anteroposterior view of the Bösch procedure. Once the osteotomy is achieved, a 2.0 mm K-wire is inserted from the toe, heading proximal just medial of the phalanges and metatarsal head. Once it reaches the proximal part of the osteotomy, the K-wire is introduced into the metatarsal bone in an intramedullary fashion

For the Bösch osteotomy (Fig. 1B), we also used a 2 mm by 15 mm Shannon burr. This osteotomy also started on the dorsal part of the metatarsal at the level of the neck of the bone. In this technique, the direction moved gradually plantar and proximal so the osteotomy was diagonal. Once it was achieved, a 2 mm K-wire was inserted, starting from the medial corner of the nail of the big toe and heading proximally, just medial of the phalange and metatarsal head. The K-wire was inserted into the metatarsal bone, proximal to the osteotomy, in an intramedullary fashion (Fig. 2).

#### Bandaging

The feet were bandaged using specifically designed dressings as described by de Prado [6], which helped maintain the correction achieved by the osteotomies.

### Radiological assessment

Fluoroscopy imaging was obtained during the procedures with a C-arm and saved in a digital format. All the measurements were made later by an external radiologist.

**Fig. 3:**
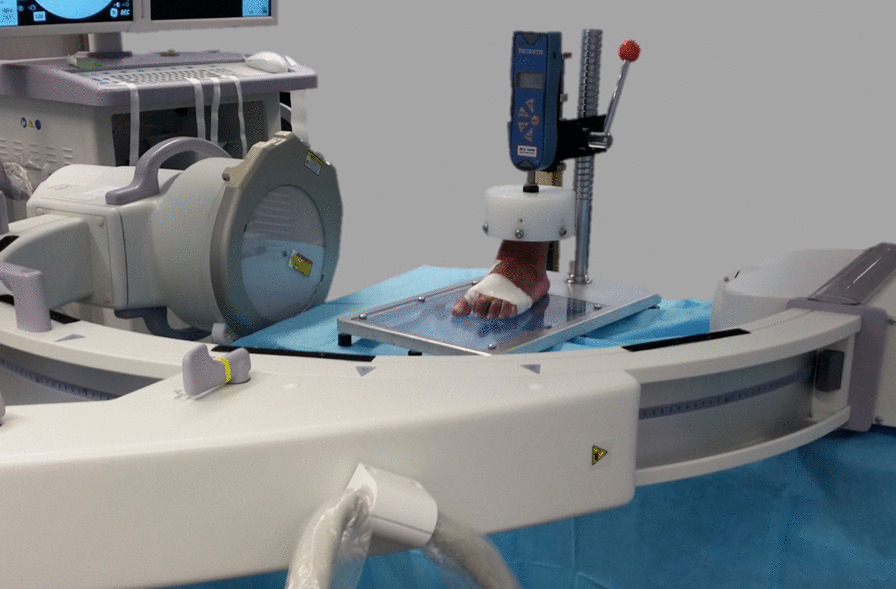
Torque-based force bench. Its design allowed a stable attachment of the tibia to be made. A vertical force could be applied and measured by a dynamometer (Mecmesin Ltd, Slinfold, United Kingdom)

In order to take fluoroscopic images, the foot was attached to a torque-based force bench (Fig. 3). Two perpendicular trans-osseous screws were used to firmly hold the distal third of the tibia to the bench. This fixed position held the ankle in a neutral position, and the medial part of the foot aligned to the bench metal side. A metallic peg was attached to the bench just anterior to the tip of the toes, to avoid forward translation of the foot when the axial force was applied. The C-arm was placed in a lateral X-ray position, parallel to the bench surface. The correct positioning of the C-arm was checked on the lateral X-ray image and the image of both bench rims was super-imposed.

Lateral fluoroscopic images of the feet were taken before the surgery and after surgery and bandaging. Afterward, using the torque-based force bench, axial weight was loaded on to the foot, progressing in 10 kg increments up to 60 kg. Lateral X-rays were taken at every 10 kg increase.

**Fig. 4:**
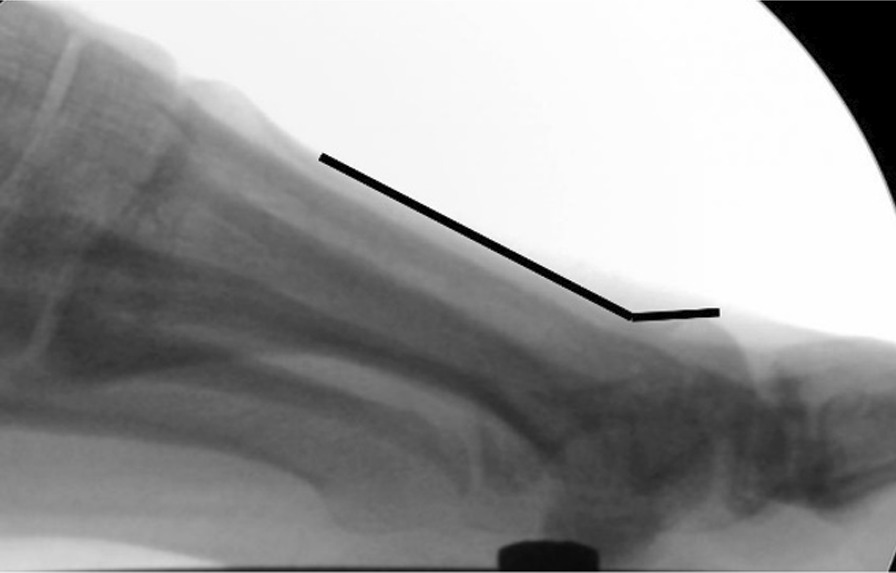
Measurement of metaphyseo-diaphyseal angle on lateral X-ray

Two parameters were measured on the lateral images:

1. Metaphyseo-diaphyseal angle (MDA), formed between two lines:
  *Diaphyseal line*: It follows the dorsal cortex of the first metatarsal diaphysis, up to the metatarsal’s neck.
  *Metaphyseal line*: It starts at the indentation of the bone’s neck and follows the dorsal cortex of the metatarsal head (Fig. 4).
2. Distance between the osteotomy fragments, measured in millimeters. From the most proximal point of the metaphyseal line, a line is traced perpendicular to the diaphyseal line.

**Fig. 5:**
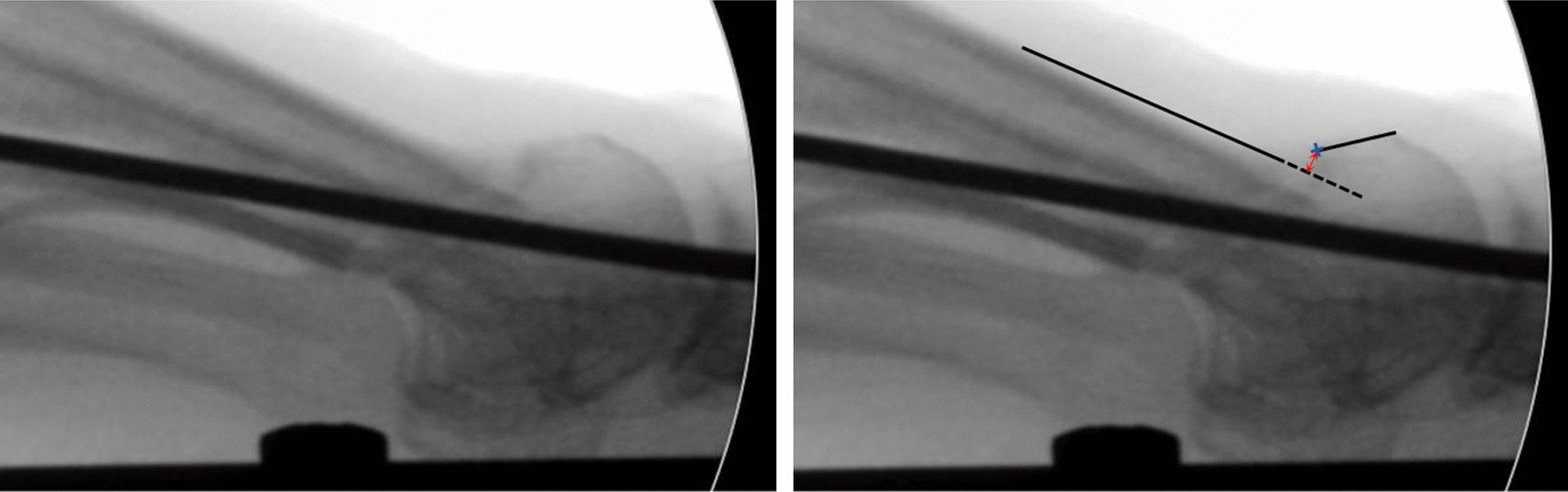
Method used to measure fragment displacement on lateral X-ray. On the left, dorsal displacement of the metatarsal head can be observed in a lateral X-ray of a Bösch osteotomy. On the right, the same X-ray with the displacement measurement. A line is drawn following the metatarsal diaphysis (continuous black line), which continues distal to the osteotomy (dashed line). A line perpendicular to the diaphyseal line is drawn (red line) from the start of the metaphyseal fragment (blue cross). This measures the distance between fragments

The point where it crosses the diaphyseal line defines the distance between the osteotomy fragments (Fig. 5).

### Statistical analysis

Shapiro–Wilk test was used to check normality. Significant differences between the two techniques either in MDA or in the distance between osteotomy fragments distance were assessed using a Student t test for all loads and for every 10 kg axial load increase.

The significance threshold was set at a two-sided alpha value of 0.05. SPSS software (SPSS 20 for Mac, IBM, Chicago USA®) was used for all analyses.

## Results

The MDA decreased in both surgical techniques, meaning that the metatarsal head tilted plantarly. The mean plantar tilt was −6.90 degrees (SD = 10.251) for chevron osteotomy and −5.34 degrees (SD = 16.621) for Bösch osteotomy. There was no significant difference between the techniques (*p* = 0.41). Progressively increasing the load on the foot did not change this tendency (*p* = 0.553) (Table 1).

**Table 1:** Changes in metaphyseo-diaphyseal angle measured in degrees on the lateral X-ray view. The results are shown in degrees

Axial load (kg)	Bösch	Chevron	Significance
10	−0.8 ± 17.6	−7.3 ± 9.6	P 0.453
20	−4.3 ± 15.7	−8.4 ± 7.1	P 0.516
30	−4.1 ± 17.8	−8.3 ± 8.1	P 0.517
40	−4.7 ± 16.4	−9.1 ± 8.1	P 0.458
50	−6.5 ± 12.8	−9.2 ± 11.6	P 0.628
60	−6.8 ± 13.3	−7.8 ± 12.9	P 0.867

**Fig. 6:**
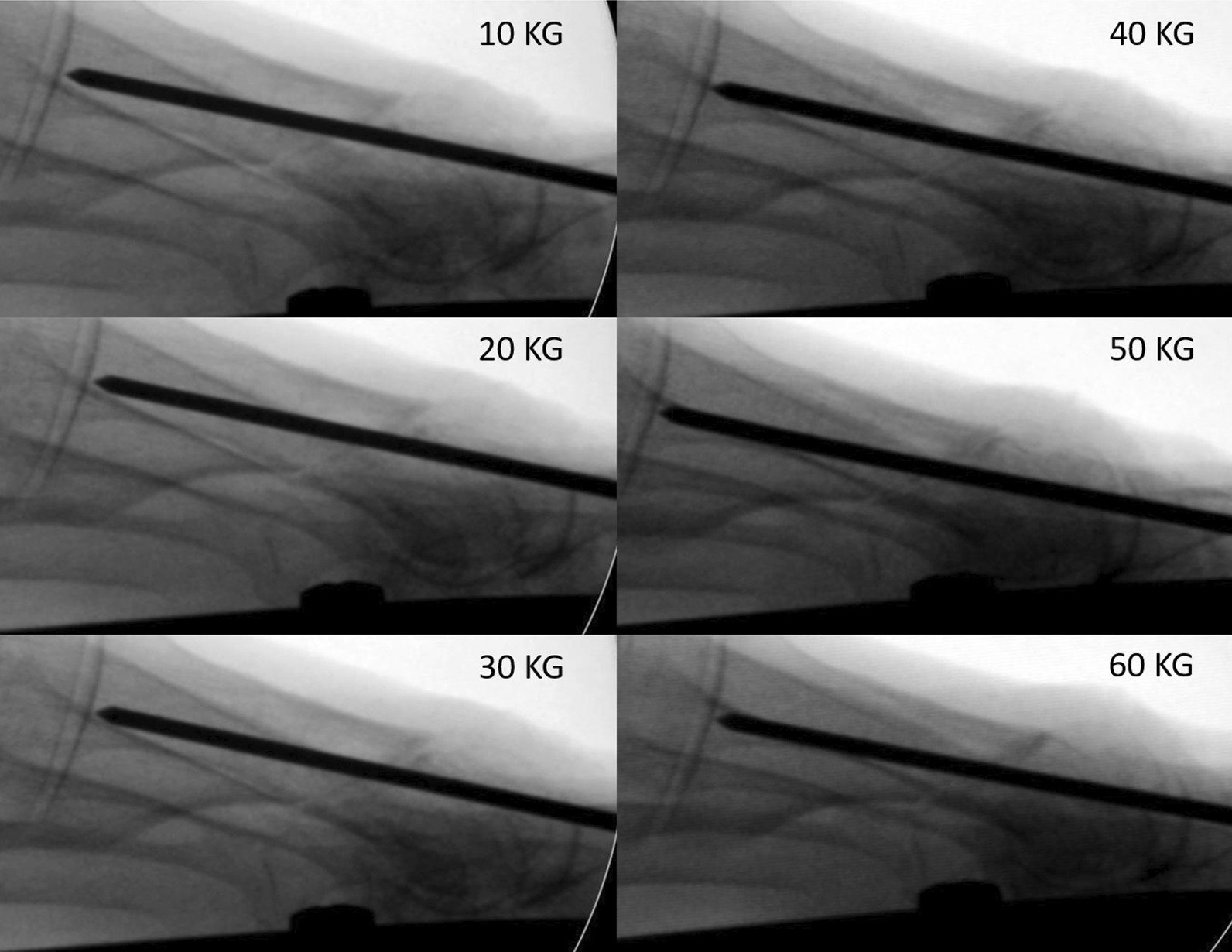
Evolution of a Bösch osteotomy under progressive axial load on the foot. Note the plantar displacement of the metatarsal head at 10 kg load, which is progressively reduced as the head is displaced dorsally when the axial load is increased. At 60 kg load, the position of the head is almost anatomical

Regarding the distance between bone fragments, Bösch osteotomy produced more plantar displacement than chevron osteotomy, which was statistically significant for the 10 and 20 kg loads (*p* = 0.031 and 0.04 respectively) (Table 2). As the load on the foot increased, the metatarsal head moved upward in the Bösch technique, so the plantar displacement decreased (Fig. 6). At loads ≥ 30 kg, the bone fragment distance did not differ significantly between the techniques (*p* = 0.114), though chevron osteotomy remained more stable throughout the load test.

**Table 2:** Distance between bone fragments, in millimeters, measured on the lateral x-ray view

Axial load (kg)	Bösch	Chevron	Significance
10	−4.3 ± 4.2	0.5 ± 1.8	P 0.031
20	−2.9 ± 0.8	0.3 ± 1.3	P 0.04
30	−3.2 ± 3.5	−0.7 ± 2.8	P 0.114
40	−2.5 ± 3.3	−0.5 ± 2.8	P 0.150
50	−2.2 ± 3.2	−0.6 ± 2.5	P 0.227
60	−2.2 ± 3.6	−0.1 ± 2.3	P 0.143

## Discussion

Hallux valgus is a frequent deformity of the first digit of the foot, with progressive abduction and pronation of the first phalanx. There is adduction, elevation, and pronation of the first metatarsal (metatarsal primus varus) concurrent with lateral capsule retraction of the metatarsophalangeal (MTP) joint [8]. In addition to its esthetic effect, hallux valgus changes the foot dynamics, leading to what Viladot defined as a first ray insufficiency [15]. Inflammation of the bursa overlying the medial eminence of the metatarsal head causes pain and discomfort, and irritation of the dorsal medial cutaneous nerve of the hallux [6, 17]. First radius insufficiency can also lead to second and third metatarsal head overload [18, 19]. Therefore, when surgery is indicated, it is essential to correct the metatarsus primus varus as well as the hallux valgus [20]. For moderate hallux valgus (intermetatarsal –IMTT- angle < 20 degrees), this can be achieved with a DFMO [3]. For greater deformities, other techniques such as proximal osteotomy of the first metatarsus can be indicated [21].

Many open surgery procedures have been described for osteotomy of the distal first metatarsal, but not until the 1980s did minimally invasive and percutaneous procedures start to appear [22]. The authors believed that the benefits from percutaneous hallux valgus surgery (esthetic improvement, soft tissue conservation, shorter surgical time, shorter recovery time, and better post-operative pain control) outweigh the potential risk of neurovascular or tendon injury, which is minimal [23]. The first widely disseminated percutaneous procedure, published by Bauer et al. [8], was a variation of Reverdin osteotomy (medial closing wedge osteotomy at the metatarsal distal third) together with an Akin osteotomy, adductor tenotomy and bunionectomy [6]. None of the osteotomies was fixated with hardware. This procedure corrects both the Hallux Valgus angle (HVA) and MDA but does not improve the IMTT angle, and it is not recommended for cases with IMTT angle greater than 12–13 degrees [5, 24].

To achieve greater IMTT angle correction, Bösch et al. [11] designed a new DFMO, not taking a wedge but using a Kirschner wire as a lever to help move the metatarsal head laterally. This procedure was later popularized by Giannini et al. [3] as Simple, Effective, Rapid and Inexpensive technique (SERI). Giannini et al. [3] published some very good radiological and functional results, although they described dorsal displacement of the metatarsal head in a very few patients (1%). To avoid this, they recommended a thick K-wire (2.0 mm). However, as the K-wire does not traverse the metatarsal head, its thickness is not necessarily key to the stability of the osteotomy, and SERI should be considered a non-fixated technique. Other authors performing SERI have found much greater metatarsal head dorsal displacements (12–20%) [25, 26], even up to 60% [4]. This displacement could lead to shortening of the first metatarsal, which could produce secondary transference metatarsal pain.

Percutaneous chevron osteotomy was first described by Vernois and Redfern [7, 27]. It is a V-shaped osteotomy in which the first part is dorsal, short, and vertical and the second part is plantar, longer, and almost horizontal [17]. It allows the HVA, MDA and IMTT angles to be corrected. Vernois and Redfern described percutaneous chevron osteotomy with a screw fixation between the bony fragments, but we used no hardware fixation. Austin [28], who first described the chevron osteotomy during the 1960s, considered the V-shaped osteotomy inherently stable, and open surgery studies show that the chevron osteotomy confers no advantage in fragment fixation [29, 30]. For this reason, and in order to compare the stability of the osteotomy shape between Bösch and chevron osteotomies under similar conditions, we decided to perform the percutaneous chevron osteotomy without fixation.

Radwan et al. [10] compared the SERI technique with an open chevron, both fixated only with a K-wire. However, to our knowledge, no comparisons between Bösch and percutaneous chevron osteotomies have been published. Most of the studies we reviewed only focused on the AP X-ray view, measuring HVA, DMA and IMTT angle, and did not assess stability in the sagittal plane. Some authors [3, 4, 9, 31] report dorsal malunion but do not specify how they measured it. Only Faour-Martin et al. [25] specify that they measured the percentage of the transverse diameter of the osteotomy line on lateral X-ray, finding 29% of dorsiflexion on average, but this only assessed the bony displacement and did not consider angulation. We have described a method that allowed us to measure both the fragment distance and the angulation systematically on lateral X-ray. To our knowledge, this is the first study to take into account changes in MDA in the sagittal plane.

With the Bösch technique, we observed a plantar displacement of 4.3 mm and a plantar angulation of 0.8 degrees with no load. As the load on the foot increased progressively, the plantar displacement decreased (2.2 mm at 60 kg axial load), but the plantar angulation of the metatarsal head continued to increase up to 6.8 degrees at 60 kg load. This variability during the stress test shows that Bösch osteotomy is highly unstable in the sagittal plane.

With chevron osteotomy there was less fragment displacement. With no load, there was a dorsal displacement of 0.5 mm, which remained quite stable, and at 60 kg load, the displacement was a 0.1 mm. Chevron displacement was significantly less than Bösch technique when the load was less than 30 kg. However, from 30 kg, as the plantar displacement of Bösch technique decreased, this statistical difference disappeared. The 0.1 mm displacement at 60 kg load in chevron osteotomy was lower than the 2.2 mm in Bösch technique, but the difference was not significant.

On the other hand, chevron osteotomy gave 7.3 degrees plantar angulation, which remained quite constant, reaching 7.8 degrees at 60 kg load (mean 6.9 degrees over all loads). Therefore, although there was less displacement of the bony fragments, chevron osteotomy failed to control the metatarsal head angulation, even though this angulation change remained stable during the loading test.

Studies investigating both Bösch and chevron techniques typically report dorsal metatarsal head displacement [3, 22, 31]. However, we observed plantar displacement in all cases. This could be explained as follows: since the plantar displacement decreased as the load on the foot increased, allowing patients to weight-bear post-operatively probably helps to displace the osteotomy upward.

Being a study based on lateral foot X-rays, the measurements might not be as reliable as those achieved with a weight-bearing computed tomography, especially as there might be a variability in the C-arm or foot placement, and also because the assessment of a lateral X-ray might be confusing due to the overlapping with the minor metatarsal bones. Further research into the measurement of MDA and fragment displacement in the sagittal plane, in which inter-observer variability is measured, is now necessary.

Although our study could have revealed greater differences between these two surgical procedures if more subjects had been examined, the fragment angulation in the sagittal plane shows unacceptable instability in both techniques. A 5–6 degree change in metatarsal head angulation could disturb the metatarsal formula and lead to iatrogenic metatarsalgia, or potentially to limitation of the flexion–extension of the metatarsophalangeal joint. We believe both techniques could benefit from sturdier fixation such as screw fixation.

## Conclusions

In the sagittal plane, chevron osteotomy was more stable than Bösch osteotomy in terms of fragment displacement. Although Bösch technique gave greater variability in angulation during the stress test, both techniques showed increased plantar angulation of the metatarsal head. For this reason, screw fixation could be advisable.

## Data Availability

All data generated or analyzed during this study are included in this published article.

## Abbreviations

DFMO: Distal first metatarsal osteotomies
HV: Hallux valgus
HVA: Hallux valgus angle
IMTT: Intermetatarsal
MDA: Metaphyseo-diaphyseal angle
MTP: Metatarsophalangeal
